# Delayed Re-Epithelialization in Periostin-Deficient Mice during Cutaneous Wound Healing

**DOI:** 10.1371/journal.pone.0018410

**Published:** 2011-04-07

**Authors:** Takashi Nishiyama, Isao Kii, Takeshi G. Kashima, Yoshinao Kikuchi, Atsushi Ohazama, Masashi Shimazaki, Masashi Fukayama, Akira Kudo

**Affiliations:** 1 Department of Biological Information, Tokyo Institute of Technology, Yokohama, Japan; 2 Department of Human Pathology, Graduate School of Medicine, The University of Tokyo, Tokyo, Japan; 3 Department of Pediodontology, Dental School, Showa University, Tokyo, Japan; Ohio State University, United States of America

## Abstract

**Background:**

Matricellular proteins, including periostin, are important for tissue regeneration.

**Methods and Findings:**

Presently we investigated the function of periostin in cutaneous wound healing by using periostin-deficient (−/−) mice. Periostin mRNA was expressed in both the epidermis and hair follicles, and periostin protein was located at the basement membrane in the hair follicles together with fibronectin and laminin γ2. Periostin was associated with laminin γ2, and this association enhanced the proteolytic cleavage of the laminin γ2 long form to produce its short form. To address the role of periostin in wound healing, we employed a wound healing model using WT and *periostin*−/− mice and the scratch wound assay *in vitro.* We found that the wound closure was delayed in the *periostin*−/− mice coupled with a delay in re-epithelialization and with reduced proliferation of keratinocytes. Furthermore, keratinocyte proliferation was enhanced in periostin-overexpressing HaCaT cells along with up-regulation of phosphorylated NF-κB.

**Conclusion:**

These results indicate that periostin was essential for keratinocyte proliferation for re-epithelialization during cutaneous wound healing.

## Introduction

Wound healing, the tissue repair process, is accomplished through the following 3 stages: inflammation, proliferation, and remodeling [1]. In the inflammation phase, neutrophilic granulocytes infiltrate into the damaged tissues, forming granulation tissue, which infiltration is then followed by the accumulation of macrophages. In the proliferation phase, keratinocytes proliferate to allow skin resurfacing for re-epithelialization, and fibroblasts derived from mesenchymal tissues around the damaged area proliferate for dermal restoration. In the remodeling phase, keratinocytes function for epidermis maturation and wound contraction; and the fibroblasts differentiate into α smooth muscle actin (αSMA)-positive myofibroblasts [2]. These latter cells express various extracellular matrix (ECM) proteins such as collagens and fibronectin for remodeling the granulation tissue into normal tissue [3]. Keratinocyte proliferation and migration are early events in wound re-epithelialization. Keratinocytes initially respond to an epidermal defect by proliferating and migrating from the free edges of the wound. The epidermal stem cells from hair follicles are thought to originate from the hair bulge, which serves as a reservoir of keratinocytes for wound healing [4].

As for keratinocyte proliferation and migration in cutaneous wound healing, cell-surface integrins, which use specific components of the ECM as ligands to receive extracellular cues, are important for assembling collagen and fibronectin to provide a basement membrane on hair follicles for supporting laminin 5 [5]. In this regard, β1integrins control the proliferation of epidermal cells [6], and play a major role in the basement membrane remodeling and keratinocyte migration that is essential for hair follicle morphogenesis [7].

To investigate the role of the ECM architecture in keratinocyte proliferation and migration, we focused on periostin, a secretory protein that assembles fibronectin to form the fibronectin meshwork architecture that constructs the collagen matrix structure [8], [9]. Thus, periostin plays a role in the construction of the ECM architecture. In mammals, periostin is expressed in the dense connective tissues, such as periosteum, periodontal ligament, and heart valve [8], [10], [11], [12], which are constantly subjected to mechanical strains from physical exercise, mastication, and blood flow and pressure, respectively. We and others recently demonstrated that the rate of heart ruptures and death caused by acute cardiac infarction is higher in *periostin*−/− mice than in their wild-type (WT) counterpart [13], [14]. The fibronectin matrix is required for the assembly of multiple ECM proteins, such as type I collagen [15]; and periostin enhances the collagen cross-linking during fibrillogenesis [16], [17].

Although periostin was previously reported to function in fibrillogenesis in the skin [17], and recently reported to be expressed in granulation tissue in mice [18], the role of periostin in cutaneous wound healing together with further detailed examination of the periostin expression patterns in the healing wound remains to be addressed. To demonstrate the role of periostin *in vivo*, we employed an experimental wound healing model using WT and *periostin*−/− mice, and found that the wound closure was delayed in the *periostin*−/− mice. Furthermore, in an *in vitro* scratch wound assay, reduced proliferation of keratinocytes was found in these *periostin*−*/*− mice. In this study, we thus demonstrated that periostin was essential for keratinocyte proliferation for re-epithelialization during wound healing.

## Results

### Expression of periostin mRNA and protein in normal mouse and human skin

**Figure 1:**
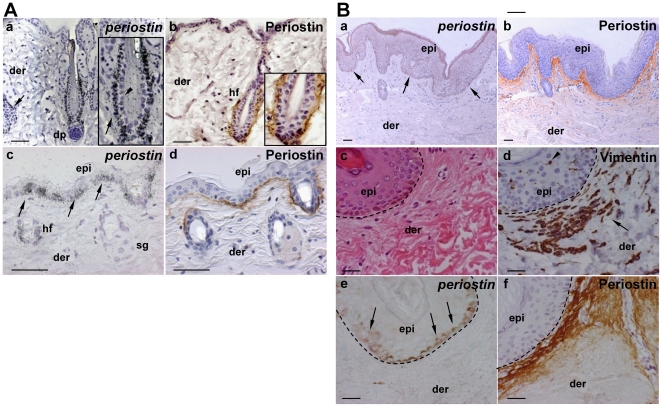
The expression of periostin in normal mouse and human skin. (A) The mRNA expression of periostin by RNA in-situ hybridization (a, c) and the protein expression of periostin by immunohistochemistry (b, d) in normal mouse skin. Insets are high-magnification views of hair follicles. Periostin mRNA was expressed in the bulge region of the hair follicle, especially in the outer cells along the basement membrane (a, arrow) not in the inner cells (a, arrowhead), and its protein was localized at the basement membrane of the follicular bulge region (b, brown stain). In the epidermis, periostin mRNA was expressed in a patch-wise pattern in the cells along the basement membrane (c, arrow), and its protein was localized at the basement membrane (d, brown stain). Scale bars: 100 µm; epi: epidermis; hf: hair follicle; sg: sebaceous gland; der: dermis (B) The mRNA expression of periostin assessed by *in situ* hybridization (a, e); and the protein expression of periostin, by immunohistochemistry (b), together with H&E staining (c) and vimentin protein expression (d) in normal human skin. Figures c -f show a high magnification of the border area of the epidermis and the dermis. Immunohistochemistry was performed using anti-periostin antibodies (b, f) and anti-vimentin antibodies (d). Periostin mRNA was expressed only in the keratinocytes (arrows in “a” and “e”) on the basement membrane, but not in melanocytes (arrowhead in “d”) or fibroblasts (arrow in “d”), and periostin protein was localized in the basement membrane (b and f, brown stain). Scale bar: 50 µm; epi: epidermis; der: dermis.

Firstly, we examined the periostin mRNA expression in normal mouse skin by performing mRNA *in situ* hybridization, and found that periostin mRNA was expressed in the bulge region of the hair follicle, especially in the outer cells along the basement membrane (Fig 1Aa, arrow) not in the inner cells (Fig. 1Aa, arrowhead), and its protein was localized at the basement membrane of the follicular bulge region (Fig. 1Ab, brown stain). In the epidermis, periostin mRNA was expressed in a patch-wise pattern in the cells along the basement membrane (Fig. 1Ac, arrow), and its protein was localized at the basement membrane (Fig. 1Ad, brown stain). Interestingly, periostin was localized in only a part of the epidermis, suggesting that periostin may function in regeneration of the epidermis. In human specimens, similarly to the mouse ones, periostin mRNA was expressed only in the keratinocytes (arrows in Fig. 1Ba and e) on the basement membrane, but not in melanocytes (arrowhead in Fig. 1Bd) or fibroblasts (arrow in Fig. 1Bd), which cell types were detected by vimentin antibody staining. Furthermore, periostin protein was localized along the basement membrane (Fig. 1Bb and f; brown stain). Since keratinocytes on the basement membrane proliferate, these results suggest that periostin may induce keratinocyte proliferation.

### Expression of periostin during the wound healing in mice

**Figure 2:**
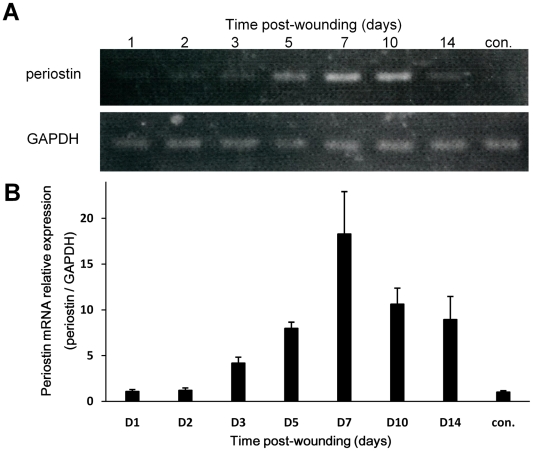
Periostin expression during wound healing. The quantitative RT-PCR for mouse periostin mRNA were performed. The wounds were removed at 1, 2, 3, 5, 7, 10 or 14 day after wounding. We used intact skin as a control tissue (con.). The representative result of RT-PCR bands is shown (A), and the quantitative result from 4 independent experiments is shown in a graph (B). Significant periostin expression was observed at day 3, and peaked at day 7. *Bars* represent the mean ± S.E.

**Figure 3:**
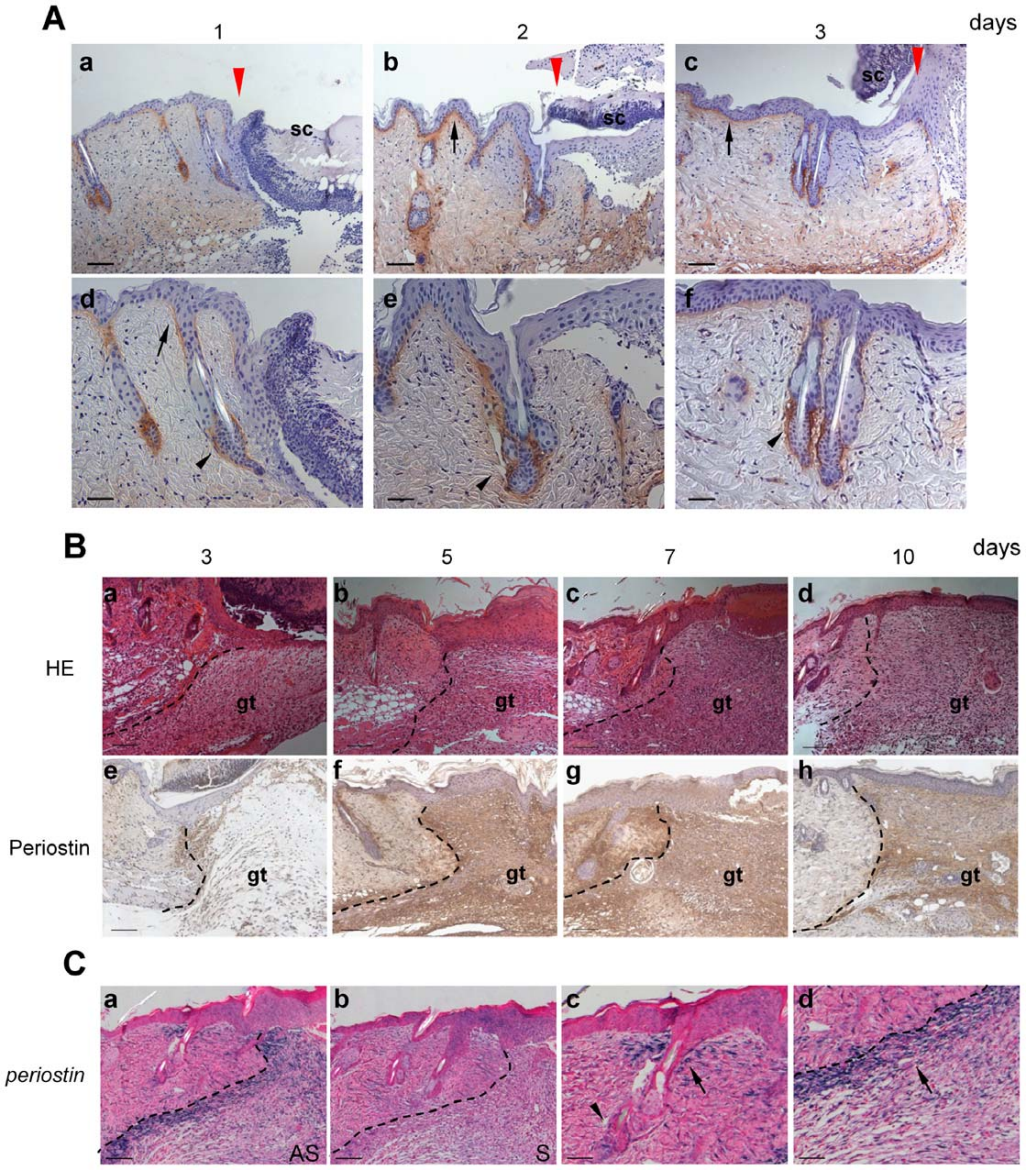
Periostin protein expression in wound healing. (A) Periostin protein expression in early wound healing. Wounds at day 1(a, d), 2(b, e), and 3(c, f) after wounding were stained with anti-periostin antibodies. Figures d, e, and f show a high magnification of “a,” “b,” and “c,” respectively. At day 1, periostin protein was expressed mainly in hair follicles (d, arrow) and weakly at the basement membrane in the hair follicle opening area (d, arrowhead). At days 2 and 3, the protein was localized more clearly at the basement membrane of the epidermis (b, c; arrow) and diffusely around the hair follicle (e, f; arrowhead). Red arrowheads indicate the wound edge. Scale bar: 100 µm (a, b), 50 µm (c, d); sc: scab. (B) H&E staining (a–d) and periostin protein expression in wounded skin assessed by immunohistochemistry (e–h) at 3, 5, 7, and 10 after wounding. Periostin was localized in granulation tissues. The dotted line in each photo indicates the border between the dermis and the granulation tissue. Scale bar: 100 µm; gt: granulation tissue (C) Periostin mRNA *in situ* hybridization of skin at 5 days after wounding. The sections were probed with the anti-sense cRNA probe (a) and the control sense probe (b). The periostin-positive area in “a” is magnified in “c and “d”: periostin-positive fibroblasts surrounding a hair follicle (arrowhead in “c”) and at the border of the wound area (arrow in “d”) are seen. The signals were not detected with the sense probe (b). Scale bar: 100 µm (a, b), 50 µm (c, d).

To detect the mRNA expression of periostin during the wound healing, we performed quantitative RT-PCR analysis of the wound area on days 1, 2, 3, 5, 7, 10, and 14 after wounding. The representative result of RT-PCR bands is shown in Figure 2A, and the quantitative result from 4 independent experiments is shown in a graph (Fig. 2B). Transcription of the periostin gene was transient, first appearing around day 3, increasing on day 5 and 7, and decreasing on day 10 (Fig. 2B), suggesting that periostin was highly expressed at the time of active wound closure, consistent with a previous report demonstrating that the granulation tissue begins to be organized on day 3 during wound healing [19]. In addition, this transient expression pattern of periostin mRNA is similarly observed in embryonic heart [12]. To confirm the protein expression of periostin on days 3, 5, 7 and 10 after wounding, we immunostained histological sections with anti-periostin antibodies (Fig. 3A). Firstly, we examined the periostin expression in early wound healing at day 1, 2, and 3 after wounding. At day 1, periostin protein was expressed mainly in hair follicles (Fig. 3Aa and arrow in d) and weakly at the basement membrane in the hair follicle opening area (Fig. 3Aa and arrowhead in d). At days 2 and 3, the protein was localized more clearly at the basement membrane of the epidermis (Fig. 3Ab, c; arrow) and diffusely around the hair follicle (Fig. 3Ae, f; arrowhead). Next, we examined the wounded skin at 3, 5, 7, and 10 after wounding (Fig. 3B). The results indicated that strong expression of periostin protein occurred at the wound border on day 3. At days 5, 7 and 10, the protein was widely found in the whole area of the granulation tissue, consistent with data recently reported [18]. Furthermore, we examined which cells expressed periostin transcripts by performing *in situ* RNA hybridization of histological sections of the wound area on day 5 and thereby detected periostin transcripts at the border between the non-wound area and wound area in the granulation tissue (Fig. 3C), similar to the protein expression area. Moreover importantly, periostin transcripts were observed in keratinocytes at the entrance of hair follicles (Fig. 3Cc, arrow), in keratinocytes around the bottom of hair follicles (Fig. 3Cc, arrowhead), and in fibroblasts at the wound edge (Fig. 3Cd, arrow). Although during wound healing periostin was expressed in both granulation tissues and keratinocytes as well as in fibroblasts at the wound edge, we focused further examination on periostin action in keratinocytes and fibroblasts for re-epithelialization; because we wanted to investigate the function of keratinocytes in hair follicles.

### Delayed wound closure in *periostin*−/− mice

**Figure 4:**
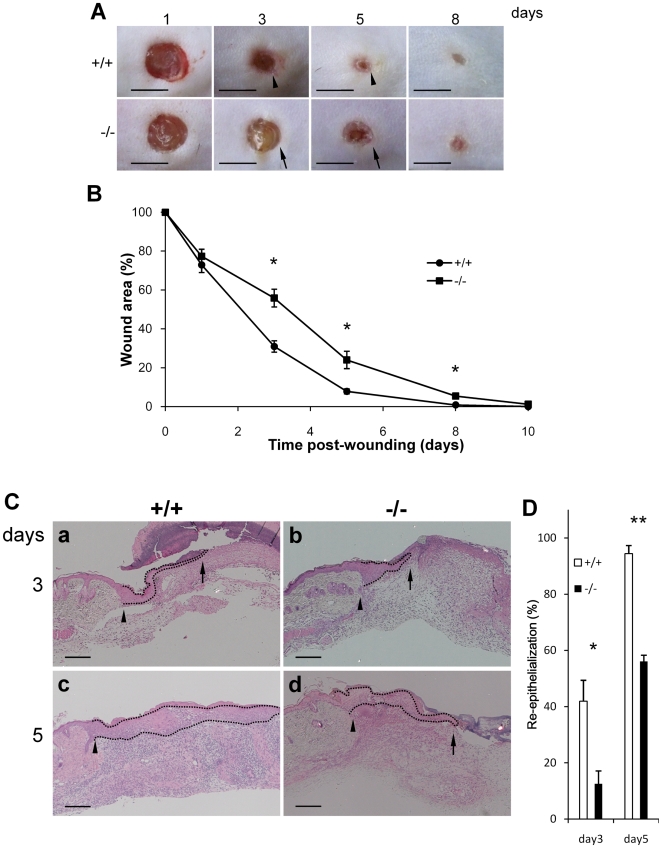
Delayed wound contraction in periostin−/− mice. (A) Representative macroscopic views of skin wounds in the periostin WT (+/+, upper row) and −/− (lower row) mice on 1, 3, 4, and 8 days after wounding. This wound healing was monitored by taking digital photographs. Scale bar: 3 mm. Note the delay of wound healing in the −/− mice. The arrow in photos of days 3 and 5 of −/− mice shows a clearer border of the wound area compared with the border of that in the +/+ mice at 3 and 5 days (arrowhead). (B) The wound size is shown. Time-course of changes in wound closure ratio after wounding on 0, 1, 3, 5, 8 and 10 days. From the photographs, percent wound size was calculated by measuring the wounded area. A total of 20 wounds were assessed (*Vertical lines* represent the mean ± S.E. *P<0.005). (C) Delayed re-epithelialization in *periostin*−*/*− mice. The wound area of WT (a, c) and *periostin*−/− (b, d) mice was excised on day 3 (a, b) or 5 (c, d) after wounding, sectioned, and stained with H&E. Arrowheads and arrows indicate the original wound edge and leading edge of the re-epithelialized area, respectively. The dotted area indicates the newly formed epidermis. Scale bar: 50 µm. (D) From the photographs, the percent re-epithelialization level of WT (a, c) and *periostin*−/− (b, d) mice was calculated by measurement of the re-epithelialized area at days 3 and 5 after wounding. Four wounds were assessed. *Bars* represent the mean ± S.E. *P<0.05, **P<0.01.

To investigate the effect of periostin on the process of re-epithelialization, we performed experimental wound healing in mice. The architecture of the skin was histologically identical between the wild-type (WT) and periostin-deficient (−/−) mice before cutaneous wounding (data not shown). Then, 4 full- thickness wounds (3 mm in diameter) were generated on the shaved back of 8-week-old mice by using a biopsy punch, and then we monitored the wound area for 10 days macroscopically. The wound tissues at days 1, 3, 5, and 8 in WT and periostin−/− mice are shown in Figure 4A, and the quantitative measurement of the level of wound closure is given in Figure 4B. On day 3 after wounding, the areas of the wounds in both WT and *periostin*−*/*− mice were decreased to 31.0±2.91 and 55.9±4.57%, respectively, of the initial area of the wounds at day 0. On days 5 and 8, these values for the *periostin*−*/*− mice, whose wounds were less contracted, were 24.0±4.46 and 5.46±1.00%, respectively; in contrast, those for the WT mice, whose wounds were more contracted, were 7.86±1.26 and 0.89±0.45%, respectively. Moreover, on day 8, the wound closure in the WT mice was almost completely accomplished; whereas in the *periostin*−*/*− mice, almost complete closure did not occur until day 10. These results demonstrate that the wound closure was delayed in *periostin*−*/*− mice, which would have been caused by defective re-epithelialization and granulation tissue formation. As to the defective re-epithelialization, as shown in Figure 4A, on days 3 and 5 in the *periostin*−*/*− mice, the border of the wound area was clearly seen (arrows in Fig. 4A), compared with that in the WT mice (arrowheads in Fig. 4A), suggesting delayed re-epithelialization in *periostin*−*/*− mice.

### Delay of re-epithelialization in *periostin*−/− mice

Since Figure 4A showed a delay in re-epithelialization in wound healing in the *periostin*−*/*− mice, we evaluated the level of this delay by performing H&E staining of tissue sections from the wound area of *periostin*−*/*− mice and WT mice at days 3 and 5 after wounding (Fig. 4C, D). The re-epithelialization ratio was determined by measuring the distance between the edge of the original wound (Fig. 4C, arrowhead) and the leading edge of the re-epithelialized area (Fig. 4C, arrow). The quantitative data is shown in graphic form (Fig. 4D). The results showed that on days 3 and 5, re-epithelialization was delayed in the *periostin*−*/*− mice. Although the re-epithelialization was nearly complete in the WT mice on day 5, it was not finished yet (around 60%) in the periostin−/− mice. Taken all together, our results indicate that the re-epithelialization after wounding was delayed in the periostin −/− mice, suggesting that periostin functions in this process.

### Reduced proliferation of keratinocytes around hair follicles in *periostin* −/− mice

**Figure 5:**
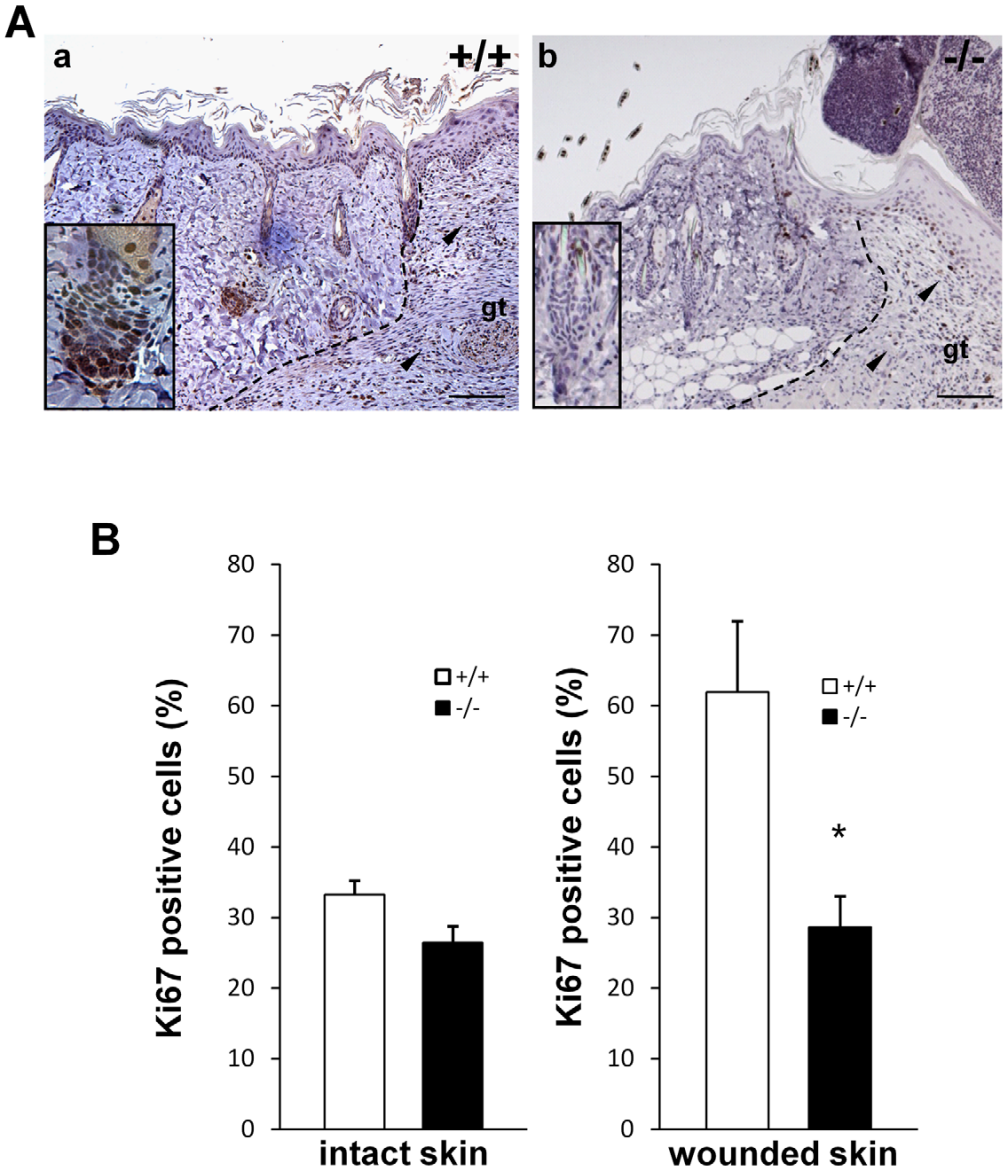
Reduced number of Ki67-positive cells in hair follicles in *periostin*-/- mice. (A) These sections of a day-3 wound in WT (+/+) (a) and *periostin*−*/*− mice (b) were stained with anti-Ki67 antibody. Insets show high-magnification views of a hair follicle. The cells in the granulation tissue of both +/+ and *periostin*−*/*− mice were positively stained with anti-Ki67 antibody (arrowhead), but those in the hair follicle of *periostin*−*/*− mice were weakly stained (inset). The dotted line indicates the border between the dermis and the granulation tissue. Scale bar: 50 µm, gt; granulation tissue (B) Ki67-positive cell index (percentage of positive cells among all keratinocytes) for the bulge region of hair follicles of intact skin or of wounded skin. A total of 6 hair follicles surrounding the wound were assessed. *Bars* represent the mean ± S.E. *P<0.05.

To further investigate the role of periostin in re-epithelialization, we examined the proliferation of keratinocytes by using anti-Ki67 antibody, which detects the prototypic cell cycle-related nuclear protein ki67, a proliferation marker, on day 3 after wounding, as shown in Figure 4C. The result showed that in the area of the granulation tissue and in the proliferative area of migrating keratinocytes, a similar number of Ki67-positive cells was observed in both *periostin*−*/*− mice and WT mice, thus indicating no significant difference in proliferation (Fig. 5). However, in the hair follicle area of the wounded skin, the number of Ki67-positive cells in the *periostin*−*/*− mice was clearly decreased to about one-half compared with that for the WT mice (see insets in Fig. 5A and right graph in 5B); whereas in the area of intact skin, no significant difference in the number of Ki67-positive cells was observed (Fig. 5B, left). These results suggest that periostin expressed in keratinocytes on the basement membrane around the hair follicles, as was shown in Figure 1A, induced the proliferation of keratinocytes to enhance the re-epithelialization.

### Keratinocyte scratch assay *in vitro*

To assess the function of periostin in the keratinocyte proliferation during the wound repair process, we employed the recently developed *in vitro* scratch assay using human keratinocyte cell line HaCaT [20]. To perform the experiment using this assay in a manner allowing comparison with the *in vivo* data from periostin −/− mice, we should carry out an siRNA experiment. However, because periostin is not significantly expressed in HaCaT cells, we employed the over-expression of periostin as a second choice.

**Figure 6:**
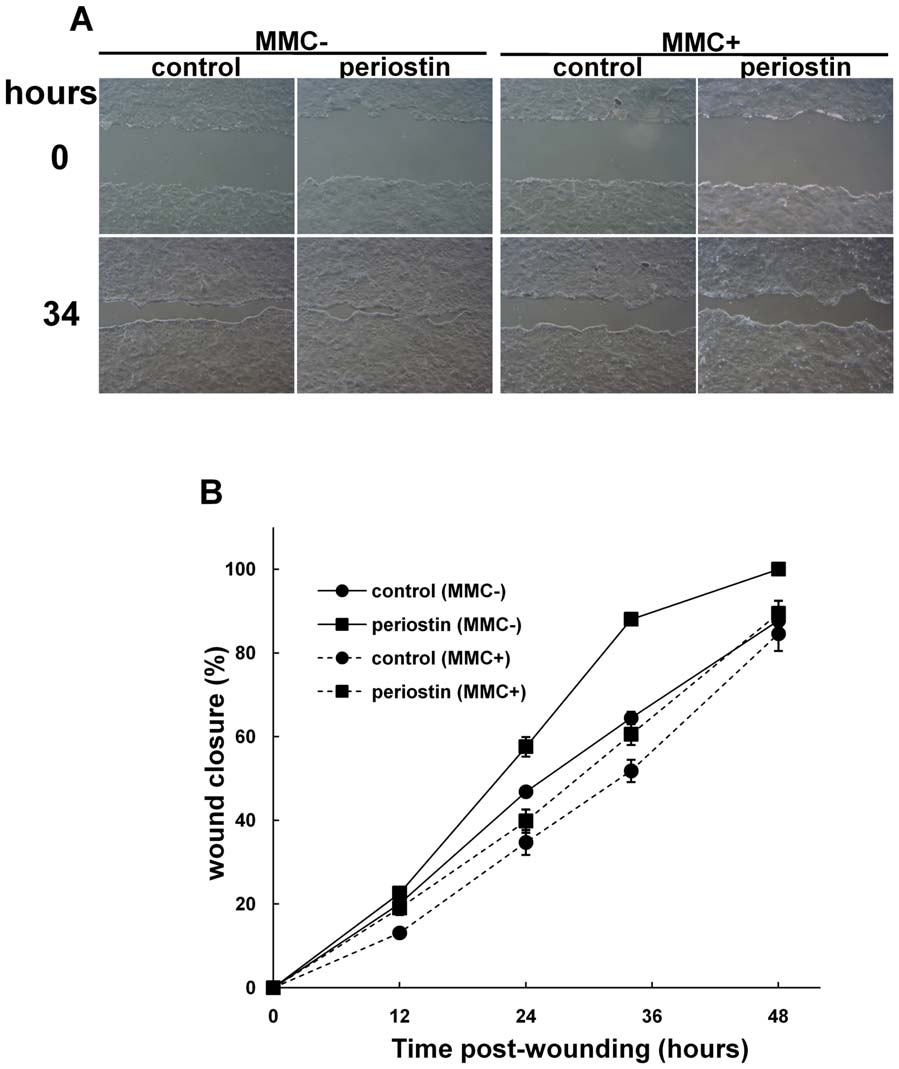
Periostin enhances re-epithelialization by inducing cell growth *in vitro* in the keratinocyte scratch assay. (A) Cells of the human keratinocyte cell line HaCaT were transfected with the periostin-HA construct, and the resulting transfectants were tested by performing the keratinocyte scratch assay to determine the effect of periostin on the wound closure. This assay was performed in the presence or absence of MMC (mitomycin C) to detect the effect of cell proliferation on the wound closure. Representative wound-closing cells (34 hr) after onset of scratching (0 hr) are shown. (B) The level of wound closure was examined at 0, 12, 24, 34 and 48 hr, after scratching the cell sheet. *Vertical lines* represent the mean ± S.E.

We transfected HaCaT cells with a mouse periostin-HA construct and used the resulting transfectants for the experiment on wound closure (Fig. 6). Before testing the wound closure, we examined the proliferation rate of the periostin-HaCaT transfectant and found no significant difference in the proliferation level between it and the cells transfected with the control vector (Figure S1). Then, we examined the extent of wound closure in this assay at 12, 24, 34, and 48 hr after scratching the cultures in the presence or absence of mitomycin C, which is an inhibitor of DNA synthesis. The onset of wound closure just after the scratching of the cell layer is shown in Figure 6A; and 34 hr later, the scratched area was filled in by the periostin-, but not by the control vector-, transfectants. The time course for wound closure was examined, and the data are shown in Figure 6B. Without mitomycin C, periostin-over-expressing keratinocytes showed a higher level of wound closure (88.0±1.25 %) than the control ones (64.5±1.48 %) at 34 hr post scratching. In the presence of mitomycin C, the level of wound closure in the periostin-over-expressing cultures at that time was slightly higher (60.5±2.51 %) than that in the control cultures (51.8±2.65 %), indicating that periostin did not significantly enhance cell migration. Taken together, these data indicate that periostin mainly induced the keratinocyte proliferation in this scratch assay, which would have a positive effect on wound closure.

### Enhancement of keratinocyte proliferation

**Figure 7:**
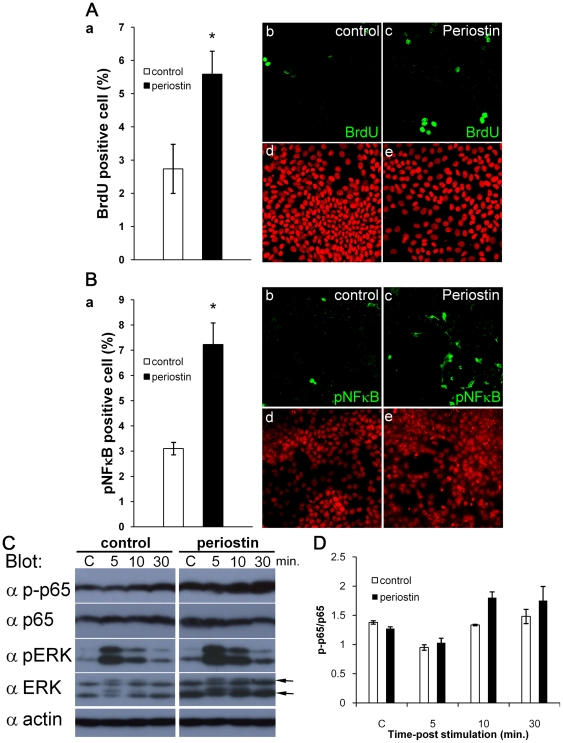
Periostin induces cell proliferation via the NF-κB signaling. HaCaT transfectants of the periostin-HA expression vector or control vector were cultured to confluence, and the cultures were continued for 1 week in 4% FBS medium. Then, the cells were serum-starved for 24 hr. After that, the cells were stimulated with 10% FBS plus 50 nM BrdU for 12 hr (A) or with 10% FBS for 15 min (B). (A) The graph shows the mean percentage of BrdU-positive cells from 5 experiments. Representative photos of BrdU signals in control and periostin-HA transfectants are also shown (x400). The lower photos show the nuclear staining by PI (propidium iodide). Note that the number of BrdU-labeled cells was increased (about 2 fold) in the periostin-HA transfectants. *Bars* represent the mean ± S.E. *P<0.05. (B) The graph shows the mean number of phosphorylated NF-κB (pNF-κB: p-p65)-positive cells from 5 experiments, in which immunohistochemistry with anti-pNF-κB antibody was performed. Representative photos of pNF-κB signals in control and periostin-HA transfectants are shown (x400). PI-positive nuclei are seen in the lower photos. Note that the number of pNF-κB-positive cells was increased (about 2 fold) in the periostin-HA transfectant. *Bars* represent the mean ± S.E. *P<0.05. (C) NF-κB phosphorylation assessed by Western blotting of HaCaT transfectants. Cells were treated with FBS in the same manner as described in “B,” then sampled after 5, 10, and 30 min after FBS treatment, and blotted with anti-pNF-κB (phosphorylated p65), anti-NF-κB (non-phosphorylated p65), anti-phospho ERK (upper signal of pNF-κB), anti-ERK, and anti-β-actin antibodies. “C” indicates the control vector. Representative data from Western blot analysis from 3 independent experiments are shown. (D) The quantitative data for the ratio of pNF-κB (phosphorylated p65) to NF-κB (non-phosphorylated p65) obtained from 3 independent experiments are shown in this graph. *Bars* represent the mean ± S.E. In the periostin- over-expressing cells, NF-κB phosphorylation and the amount of ERK (arrows in “C”) were increased.

To obtain direct evidence of keratinocyte proliferation, we performed the experiments on BrdU incorporation and phosphorylation of p65, which occur as the result of activation of NF-κB in the downstream of integrin β4 during epidermal growth [21] (Figure 7). HaCaT transfectants of the periostin-HA expression vector or control vector were cultured further for 1 week after confluence to generate the ECM structure *in-vitro*, serum-starved for 24 hr, then treated with BrdU for 12 hr. The representative photos of BrdU signals are shown (Fig. 7A, right). The number of BrdU-labeled cells was increased (about 2 fold) in the periostin-HA transfectants (Fig. 7A, graph, *P*<0.05). Furthermore, NF-κB phosphorylation was examined by staining with anti-phosphorylated NF-κB (pNF-κB: p-p65) antibody (Fig. 7B). The number of pNF-κB-positive cells was increased (about 2 fold) in the periostin-HA transfectant (*P*<0.05), consistent with the data on BrdU incorporation. Thus periostin enhanced the proliferation of keratinocytes. Since NF-κB phosphorylation is dependent on the binding of β4 integrin, we examined the expression of β4 integrin. The expression of β4 integrin in HaCaT cells was observed, although no significant difference in expression was found between control and periostin transfectants (Figure S2). Besides immunostaining, we examined NF-κB phosphorylation by Western blotting of HaCaT transfectants (Fig. 7C). Cells were treated with serum in the same manner as described in Materials and Methods for immunostaining, then sampled after 5, 10, and 30 min after serum treatment, and blotted with anti-pNF-κB (phosphorylated p65), anti-NF-κB (non-phosphorylated p65), anti-phospho ERK (upper signal of pNF-κB), anti-ERK, and anti-β-actin antibodies. The quantitative data for the ratio of pNF-κB (phosphorylated p65) to NF-κB (non-phosphorylated p65) is indicated graphically (Fig. 7D). In the periostin-over-expressing cells, NF-κB phosphorylation was enhanced 10 min after serum treatment. On the other hand, although ERK phosphorylation was not significantly induced, interestingly, the amount of ERK (non-phosphorylated ERK; shown by arrow in Fig. 7C) in the periostin- over-expressing cells was preferentially increased about 2 fold, compared with that of the control-vector transfectants, reflecting probably the ECM structure in the presence of a high level of periostin. Taken together, periostin induced the proliferation of keratinocytes through the phosphorylation of NF-κB.

### Enhancement of laminin γ2 proteolytic cleavage by periostin

**Figure 8:**
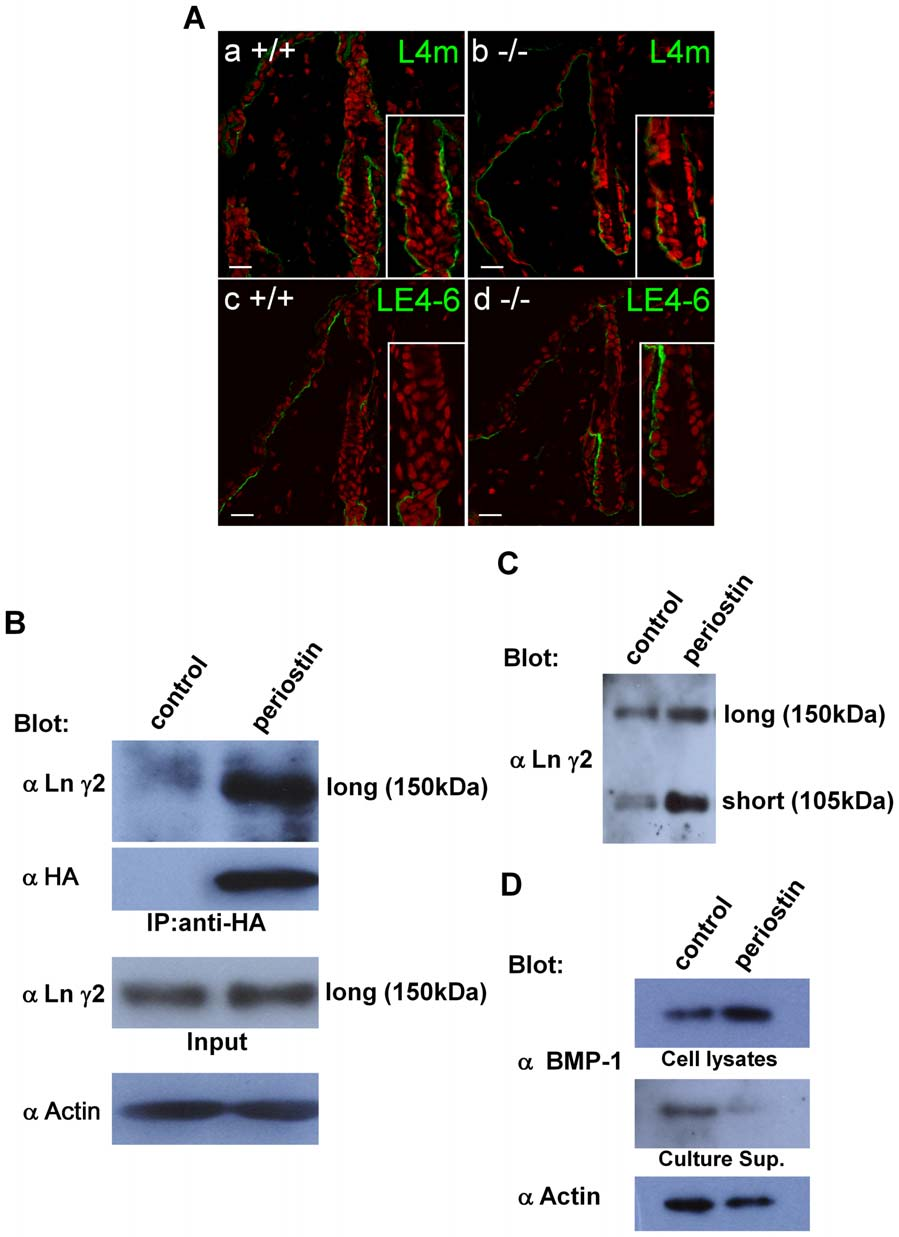
Enhancement of the proteolytic cleavage of laminin γ2 by its association with periostin. (A) Immunohistochemistry of laminin γ2 in hair follicles. Sections of WT (+/+) (a, c) and −/− hair follicles (b, d) were immunostained with 2 different anti-laminin γ2 antibodies, i.e., L4m, recognizing both the long and short forms of laminin γ2 (a, b), and LE4-6, which recognizes only the long form (c, d). The red color shows the nuclear staining by PI (propidium iodide). (B) Immunoprecipitation of periostin and laminin γ2. With anti-HA antibody periostin was immunoprecipitated from the periostin-HA HaCaT transfectants that expressed the long-form laminin γ2 (Input) and from the control cells treated with the vector only (control), and Western blot analysis was performed with anti-laminin γ2 antibody (1–97) on this immunoprecipitant. The long form of laminin γ2 was detected as well as the perostin signal. Anti-actin antibody detected actin as a control. (C) Western blot analysis of laminin γ2. The periostin-HA HaCaT transfectants highly produced the immature laminin γ2 (non-cleaved long form: 150 kDa) and the activated laminin γ2 (cleaved short form: 105 kDa), as detected by anti- laminin γ2 antibody (1–97). (D) BMP-1in cell lysates or culture supernatants (Culture Sup.) from the periostin-HA HaCaT transfectants and from the control cells treated with the vector only (control). The accumulation of BMP-1 in cell lysates from the periostin-HA HaCaT transfectants was increased. The proteins in total cell lysates and cell culture supernatants were each separated by SDS-PAGE, and blotted with anti-BMP-1; and the former were blotted with anti-β-actin antibodies.

As was shown in Figures 6 and 7, periostin had an effect on wound-dependent cell proliferation; whereas no significant change in cell proliferation was observed under the normal cell culture condition (Figure S1). Since periostin is located on the basement membrane around hair follicles, this cell proliferation induced by periostin may have been possibly caused by alteration of the microenvironment of the basement membrane, which led us to investigate a representative basement membrane protein, laminin 5, which is a trimer protein composed of α3, β3, and γ2 chain. Since in skin, laminin γ2 is preferentially expressed, and the immature long form of laminin γ2 is proteolytically cleaved to generate its short form, we examined the laminin γ2 chain [22]. To visualize laminin γ2 expression *in vivo*, we immunohistochemically examined hair follicles by using 2 different anti-laminin γ2 antibodies, i.e., L4m, which recognizes both the long and short forms of laminin γ2, and LE4-6, which recognizes only the long form. The results showed strong expression of laminin γ2 in WT mice (Fig. 8Aa), whereas a weaker expression of it was detected in the *periostin*−*/*− mice (Fig. 8Ab), suggesting that periostin may be involved in laminin γ2 expression. In contrast, the long form was scarcely detected in WT mice, but was preferentially observed in the *periostin*−*/*− mice (Fig. 8Ad, green fluorescence), suggesting that, because of the presence of the accumulated long form in the *periostin*−*/*− mice, the proteolytic cleavage of laminin γ2 was defective in these mice. To further find the relation between periostin and laminin γ2, we examined the physical interaction between these 2 proteins by using the same periostin-HA HaCaT transfectants cultured for 1week after confluence. Immunoprecipitation with anti-HA antibody-coupled beads followed by Western blot analysis using anti-HA antibody and another anti-laminin γ2 antibody (1-97) was carried out (Fig. 8B). The result showed that the long form of laminin γ2 was pulled down with periostin, suggesting the association of periostin with the immature long form of laminin γ2 on the basement membrane. To investigate the role of periostin in laminin γ2 formation, we examined supernatants of the periostin-HA HaCaT transfectants and found that the level of the short form of laminin γ2 (105 kDa) was increased, compared with its level in the control vector transfectants (Fig. 8C). This result suggests that periostin enhanced the proteolytic cleavage of laminin γ2 to produce the short form.

In recent a publication, we reported that periostin can bind to BMP-1 inside the cell but not outside the cell [16]. In previous reports, BMP-1 was shown to be a major proteinase for laminin digestion [23], [24]. Therefore, we examined BMP-1 localization in the presence of periostin. The result shown in Figure 8D indicates that BMP-1 was localized inside the cell, as it was detected in the cell lysate but not in the culture supernatant, consistent with the data previously published by Maruhashi et al. [16], who showed that periostin binds to BMP-1 inside the cell. Our result thus suggests that BMP-1 probably binds to periostin in keratinocytes for activation of BMP-1 to possibly digest laminin γ2.

Taken together, our data indicate that periostin located on the basement membrane of a hair follicle may activate BMP-1 for the proteolytic cleavage of laminin γ2 to produce its short form.

### Fibronectin localization in hair follicles

**Figure 9:**
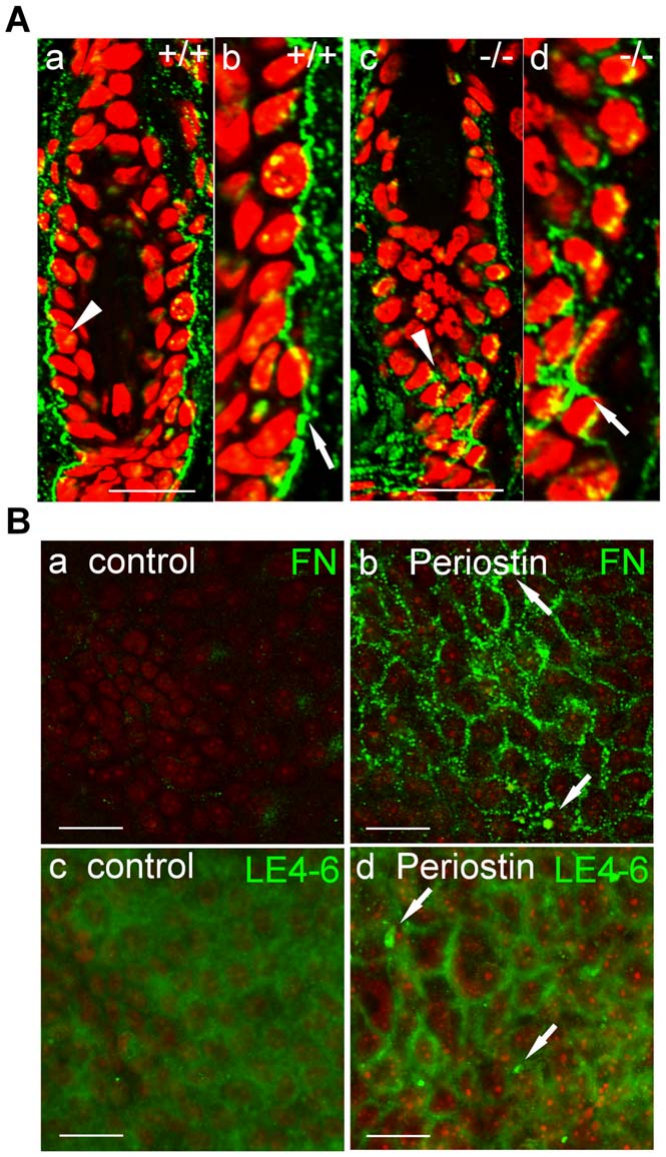
Fibronectin localization in hair follicles of WT and *periostin*−*/*− mice. (A) Fibronectin expression (green fluorescence) was examined in hair follicles from WT (+/+; a, b) and *periostin−/−* mice (c, d) by using anti-fibronectin antibody. The red color shows the nuclear staining by PI. Photos “b” and “d” show high magnification views of “a” and “c,” respectively. Fibronectin was localized mainly at the basement membrane (b, arrow) and rarely in the cytoplasm in WT mice (a, arrowhead). However, in *periostin−/−* mice, the surface expression of fibronectin was rarely detected, and preferentially observed in the cytoplasm (c, arrowhead; d, arrow). Scale bar: 20 µm. (B) Fibronectin and laminin γ2 localization on the periostin HaCaT transfectant. The localization of fibronectin and laminin γ2 proteins on the cell surface of the periostin-HA (b, d) or the control vector (a, c). HaCaT transfectants were examined by using anti-fibronectin (a, b) and anti-laminin γ2 antibody (LE4-6; c, d). Fibronectin localization in periostin transfectants shows a patched pattern (b, arrow), consistent with that for laminin γ2 (LE4-6) in the same transfectants (d, arrow). Scale bar: 20 µm.

Next, we performed immunohistochemistry to detect another ECM, fibronectin, which is involved in keratinocyte proliferation and migration, on the basement membrane of hair follicles. As a result, we observed that the signals of fibronectin for the *periostin*−/− mice were weaker and had disappeared in some areas, compared with those for WT mice (Fig. 9A). To further demonstrate the effect of periostin on fibronectin expression, we examined the localization of fibronectin and laminin γ2 proteins on the cell surface of the periostin-HA HaCaT transfectants cultured for 1 week after confluence by using anti-fibronectin and anti-laminin γ2 antibody (LE4-6, Fig. 9B). Fibronectin localization in the periostin transfectants showed a patched pattern (Fig. 9Bb, arrow), consistent with that for laminin γ2 (LE4-6) in the same transfectants (Fig. 9Bd, arrow). These results show that both fibronectin and laminin γ2 proteins were apparently located on the cell surface in a patched pattern, suggesting that periostin supported the localization of fibronectin and laminin γ2 on the basement membrane of a hair follicle for constructing some unknown structure.

## Discussion

We demonstrated that periostin functioned in keratinocyte proliferation in cutaneous wound healing, suggested by the abnormal phenotype of *periostin−*/*−* mice in the mouse wound healing model, which showed delayed wound closure coupled with delayed re-epithelialization. Skin is composed of 2 kinds of tissues, i.e., the epidermis, the cells of which form a barrier against the outside environment, and the dermis, which provides support and nutrition to the epidermis. Regarding epidermis formation, keratinocyte migration through integrin α2β1 is essential, which is activated by laminin γ2, a component of laminin 5, together with fibronectin [25]; and migration is always coupled with proliferation *in vivo*. Indeed, in integrin β1 conditional knockout mice, a severe defect in epidermal proliferation is observed [7], in which proliferation is specifically distinguished from migration. Therefore, one of the molecular mechanisms of periostin action in keratinocyte proliferation as well as in migration in wounding involves the association of periostin with laminin γ2 produced by keratinocytes. In our previous study on human cells, we showed that a positive area of periostin deposition is also strongly correlated with that of the laminin-5γ2 chain in the linear basement membrane [26]. Two forms of laminin γ2 are produced, the immature long form (150 kDa) and the short form (105 kDa), the latter being generated by proteolytic cleavage of the former. Periostin enhanced the production of the short form, suggesting that periostin may function in enhancement of proteolytic cleavage of the long form to produce the short form, consistent with a previous study on periostin revealing that it enhances the proteolytic cleavage to produce the mature form of Notch1 [27]. Moreover, it was earlier reported that bone morphogenetic protein 1 (BMP-1) is responsible for this proteolytic cleavage of laminin γ2 [23], [24]. We also recently reported that periostin is associated with BMP-1 to enhance the proteolytic activity of BMP-1 [16], which may occur in the laminin γ2 cleavage. Our present data showing BMP-1 localization in cell lysates in the presence of periostin (Fig. 8D), which agrees with our previous data [16], supports our proposition that periostin is associated with BMP-1 in keratinocytes for proteolytic digestion of laminin γ2. Furthermore, the laminin γ2 complex together with fibronectin is involved in assembly of the interhemidesmosomal basement membrane [25], in which fibronectin may be involved to form an extracellular meshwork architecture coupled with periostin [9]. In this respect, the low and disordered expression of fibronectin in *periostin−/−* hair follicle shown in Figure 9A suggests the degradation of this architecture. Further investigation by electron microscopy should reveal the structure of this complex on the basement membrane.

Regarding keratinocyte proliferation, we performed BrdU incorporation to obtain direct evidence of proliferation, and also carried out an experiment to detect NF-κB phosphorylation. Since we could not perform the double staining for BrdU and phosphorylated NF-κB because the experimental protocols for detection are different (i.e., BrdU for 12 hr, phosphorylated NF-κB for 15 min), we could not analyze whether BrdU-positive cells were also phosphorylated NF-κB-positive ones. However, we emphasize that the percentage of BrdU- positive cells (5.5%) was quite similar to that of phosphorylated NF-κB-positive cells (7%). In addition, based on an earlier study it was reported that in keratinocytes, NF-κB activation is an evidence of proliferation and that the upstream factor of NF-κB is epidermal growth factor (EGF), which enhances keratinocyte proliferation together with integrin β4 and laminin 5 [28]. Therefore, it is possible that one of the serum factors is EGF. However, in treatment of keratinocytes by EGF, no significant activation of NF-κB signals was observed (data not shown), suggesting that an unknown factor in serum can induce proliferation of keratinocytes in the presence of periostin.

Moreover, one of downstream signals of laminin 5 is p16^INK4A^ activation, a protein that induces cell motility in wound healing [29]. The histological analysis of hair follicles with anti- p16^INK4A^ antibody indicated a lower expression of p16^INK4A^ in *periostin−*/*−* mice (data not shown), which is evidence showing that laminin 5 was inactive in the *periostin−*/*−* hair follicle.

The absence of a hair follicle induces a delay in cutaneous wound healing, indicating the importance of hair follicle stem cells in wound healing [30]. Moreover, recent observations have revealed that bulge stem cells rapidly migrate towards the interfollicular epidermis in wounding to help the rapid regeneration of the wounded skin [31]. Regarding these bulge stem cells, Ito et al. reported that epithelial stem cells in the bulge region are responsible for acute wound repair [4]. Moreover, Snippert et al. reported that another type of bulge stem cell, which expresses the Lgr6 protein, can differentiate into the different cell lineages of skin epidermis [32]. During wound repair, Lgr6-expressing progenitors, which originally reside in the isthmus region of the hair follicle, migrate to repair the wounded skin. Considering the important function of bulge stem cells in wound repair, periostin, which is expressed in interfollicular cells similarly as in bulge stem cells, associates with laminin γ2 and fibronectin on the basement membrane of the hair follicle. This association might induce the migration of these cells. Furthermore, we observed the interesting patch-wise pattern of periostin expression in the epidermis, as was shown in Figure 1Ac (arrows), which is similarly found as the expression pattern of the epidermal proliferative unit (EPU) [33]. EPU contains a single stem cell in the middle of a cluster of 10 basal cells [33], suggesting that periostin may act on stem cells in the epidermis.

During wound closure, there is another important event that occurs, i.e., the formation of the granulation tissue, which is responsible for the formation of the dermis. In this event, the migration of myofibroblasts into the wound area is necessary for the wound closure and construction of the granulation tissues. Our results suggest that the delayed wound healing in the *periostin−*/*−* mice may have arisen from defective migration of myofibroblasts into the wound area, indicating the possibility of that periostin functions in myofibroblast migration and/or differentiation. Moreover, Hattori et al. reported that MMP-13, expressed in the leading edges of epidermal cells in wound healing, plays a role in the formation of granulation tissue [34]. Since it was recently reported that periostin regulates MMP-13 expression, and that its pattern is similar to that of periostin during wound healing [12], MMP-13 may also be involved in the abnormal phenotypes of *periostin −/−* mice. We plan to investigate further the function of periostin in the formation of the granulation tissue in the future.

Finally, we propose the following tentative scheme of periostin function on the basement membrane of hair follicles: Periostin acts as a scaffold protein by binding to laminin γ2, fibronectin, and the proteinase BMP-1 to activate BMP-1 function for the cleavage of laminin γ2, which is followed by induction of keratinocyte proliferation through NF-κB phosphorylation. In this scaffold model, the main player constructing this fundamental structure is fibronectin. Periostin stabilizes the fibronectin structure on the cell surface for laminin γ2 cleavage.

## Materials and Methods

### Wound healing model

To perform full-thickness skin excision, we anesthetized 8-week-old mice and took 4 equidistant 3-mm-diameter skin biopsies from a shaved area of the dorsal skin (telogen, pink skin) with a sterile disposable biopsy punch (diameter of 3 mm; Kai Industries, Tokyo, Japan). The wound was allowed to heal in the open air, and monitored by taking digital photographs at selected time points.

### Tissue specimens

Tissue specimens consisted of non-neoplastic tissues of mouse and human skin (n = 10). All human specimens were retrieved from the files of the Department of Diagnostic Pathology, The University of Tokyo Hospital. All the samples were approved for use in this research by the Ethics Committee of the University of Tokyo (No. 1220). These tissue samples had been formalin fixed and embedded in paraffin for histopathological examination.

For the histological analysis of the skin of *periostin−/−* mice [14], we used three 8-week-old mice homozygous for the disrupted periostin gene, and the same number of their wild-type littermates. Half of each tissue specimen was immediately frozen and stored at *−*80°C, and the other half was fixed with 4% paraformaldehyde (PFA) in 0.1 M phosphate buffer overnight at 4°C followed by embedment in paraffin.

### Histology and immunohistochemistry

A 4-µm paraffin section of each sample was prepared and stained with H&E. For immunohistochemistry, paraffin sections were reacted with rabbit anti-periostin antibody or anti-Ki67 antibody (YLEM, Rome, Italy) or anti-fibronectin antibody (Sigma-Aldrich, St Louis, MO) after the endogenous peroxidase had been blocked with 3% H_2_O_2_, and nonspecific binding was prevented by treatment with 2% bovine serum albumin (BSA; Nacalai Tesque, Kyoto, Japan) in Tris-buffered saline. Then they were incubated with Alexa 488-labeled goat anti-rabbit antibody (Invitrogen, Carlsbad, CA) or EnVision+ (anti-rabbit; DAKO Japan, Tokyo, Japan) and visualized by incubation with DAB+ (DAKO Japan) for 10 min. The sections were counterstained with Mayer' hematoxylin, dehydrated, and mounted.

For laminin γ2 chain immunostaining, fresh-frozen tissue sections (9 µm) were fixed with Mildform (R) (WAKO Pure Chemical, Osaka, Japan) for 20 min at room temperature. Then they were incubated with 1% pepsin (Nacalai Tesque) in phosphate-buffered saline (PBS) for 20 min at 37°C. After having been blocked with 2% BSA in TBS, the sections were reacted overnight at 4°C with anti-laminin γ2 chain antisera [35] (L4m: recognizing short and long chain, LE4-6: long form only). Then they were incubated with Alexa 488-labeled goat anti-rabbit antibody. Nuclei were stained with propidium iodide (PI; Nacalai Tesque).

### *In situ* hybridization for periostin mRNA

Antisense and sense cRNA probes were generated by *in vitro* transcription of 1.5-kb mouse periostin cDNA [8] and 2.3-kb human periostin cDNA [20] by using a DIG RNA Labeling kit (Roche, Indianapolis, IN) or S14. *In situ* hybridization for detecting the mRNA was conducted by following the manufacturer's instructions (Roche, Basel, Switzerland) and was described previously in detail [36].

### Semi-quantitative and quantitative periostin mRNA RT-PCR

The wound tissue was collected from anesthetized mice, and total RNA was isolated by the acid guanidine-thiocyanate/phenol/chloroform method. Reverse transcription was performed with a Ready To Go 1^st^ strand cDNA kit (GE Healthcare Bio-Sciences AB, Uppsala, Sweden). For semi-quantitative real-time RT-PCR for the mouse periostin gene, primers 5′-ggaattcggcattgtgggagccactacc-3′ and 5′-ggtcgactcaaatttgtgtcaggacacggtc-3′ were used. Primers 5′-actttgtcaagctcatttcc-3′ and 5′-tgcagcgaactttattgatg-3′ were used for the housekeeping gene, GAPDH, which was used as a control. For quantitative real-time PCR, primers 5′-tgctgccctggctatatgag-3′ and 5′-gtagtggctcccacaatgcc-3′ for *periostin* and primers 5′-agaacatcatccctgcatcc-3′ and 5′-cagtgagcttcccgttcagc-3′ for GAPDH were used.

### Cell culture

The human keratinocyte cell line HaCaT was cultured in Dulbecco' modified Eagle medium (DMEM, Nacalai Tesque) containing 10% fetal bovine serum (FBS, JRH Biosciences, Inc., Lenexa, KS) at 37°C in a humid 5% CO_2_ atmosphere.

### Transfection

An expression vector for mouse periostin with an HA tag was previously constructed in pCAGIPuro [20]. Cells were transfected with plasmid DNAs by using polyethyleneimine [37] (Sigma-Aldrich).

### Scratch wound assay

HaCaT cells were cultured for 1 week in DMEM supplemented with 5% FBS. For the scratch wound assay [38], the cells were serum-starved for 20 hr and incubated with or without 10 µg/ml mitomycin C (Sigma-Aldrich) for 4 hr to block proliferation. Then a cell-free area was created by scratching the monolayer with the tip of a 200-µl pipette (n  =  5). Cell migration into the wound area was monitored with the cells in DMEM supplemented with 10% FBS.

### Western blotting

For Western blotting of laminin, cells were cultured for 1 week with DMEM supplemented with 5% FBS and lysed with SDS sample buffer containing 50 mM dithiothreitol; and the lysates were then boiled for 5 min. Equal amounts of samples were size-separated on 7.5% polyacrylamide gels and electroblotted onto a polyvinylidene difluoride (PVDF) membrane. Non-specific binding was blocked by immersion of the membranes for 30 min in 5% skim milk in Tris-buffered saline (TBS) containing 0.1% Tween (TBS-T) at room temperature. The membranes were washed with TBS-T and then incubated for 1 hr at room temperature with primary antibody, i.e., monoclonal anti-mouse laminin γ2 antibody (1–97) [39], anti-HA antibody (1∶1000, Sigma-Aldrich), anti-beta actin antibody (1∶500,000, AC-15; Sigma-Aldrich), anti-β4 integrin (CD104) antibody (1∶100, ELF1; Novocastra, Newcastle, UK), polyclonal anti-phospho p65(phospho NF-κB) antibody (1∶1000, Cell Signaling Technology, Boston, MA), anti-p65 antibody (1∶2000, Santa Cruz Biotechnology, Santa Cruz, CA), anti-phospho ERK antibody (1∶1000, Cell Signaling Technology), anti-ERK antibody (1∶500, Cell Signaling Technology) or anti-BMP-1 antibody (1∶1000, Thermo Scientific, Yokohama, Japan). After this incubation, the membranes were washed again and incubated for 1 hr with secondary antibody. The antigen was detected by using ECL Western blotting Detection Reagents (Amersham Bioscience, Buckinghamshire, UK) according to the manufacturer's instructions.

### Immunocytochemistry and BrdU staining

The transfected HaCaT cells were cultured in 4% FBS/DMEM for 1 week after confluence on coverslips, and then serum-starved for 24 hr. The cells were next washed 3 times with cold PBS and subsequently fixed with fresh 4% PFA in PBS for 15 min at RT. After a wash with PBS, the cells were made permeable with 0.1% Triton X-100 for 5 min at RT, blocked with 2% BSA in TBS (pre-heated at 56°C for 30 min) for 30 min at RT, and then were incubated with primary antibody in 2% BSA in TBS at 4°C. Further incubation with Alexa-488-labeled secondary antibody and PI for 1 hr was performed, followed by washing and mounting. For primary antibodies, we used anti-phospho NF-κB antibody (p-p65; Anaspec, USA), anti-BrdU antibody (Invitrogen), anti-fibronectin antibody (LabVision Corporation, USA), and anti-laminin γ2 chain antisera (LE4-6).

For BrdU (Sigma-Aldrich) staining, cells were labeled by incubation for 12 hr after serum starvation with 50 nM BrdU dissolved in 10% FBS/DMEM; and the cells were then fixed and thereafter incubated in HCl to denature the DNA. Briefly, incubated with 1N HCl for 10 min at 4°C, 2N HCl for 10 min at RT, and then 2N HCl for 20 min at 37°C. Immediately after the acid incubations, we neutralized the cells by incubating them in borate buffer (0.1 M) for 10 min at RT.

### Immunoprecipitation

Cellular extracts were prepared with extraction buffer (25 mM HEPES-NaOH, pH 7.0, containing 100 mM NaCl and 0.5% Nonidet P-40) on ice. Clarified lysates were incubated for 1 hr with anti-HA antibody (Santa Cruz Biotechnology) and then for 1 hr with protein G-Sepharose (Amersham Biosciences) at 4°C. The bound proteins were eluted with SDS sample buffer containing 50 mM dithiothreitol.

### Statistics

Data were expressed as the mean ± S.E. Statistical significance was assessed by use of the unpaired *t* test. (*P* values <0.05 were considered significant.)

## Supporting Information

Figure S1 Proliferation of the periostin-HA HaCaT transfectant. The proliferation was compared between the transfectants produced with the periostin-HA or the vector only (control).
(TIF)

Figure S2 The β4 integrin expression in control and periostin-HA transfectants. Since NF-κB phosphorylation is dependent on the binding of β4 integrin and laminin 5, we examined the expression of β4 integrin, and observed the expression of β4 integrin in HaCaT cells; although no significant difference in expression was found in between control and periostin transfectants.
(TIF)
