# Supplementary figures and images for: Delayed Re-Epithelialization in Periostin-Deficient Mice during Cutaneous Wound Healing
Source: PLoS One. 2011 Apr 7;6(4):e18410. doi: 10.1371/journal.pone.0018410 (PMC3072397; doi:10.1371/journal.pone.0018410)

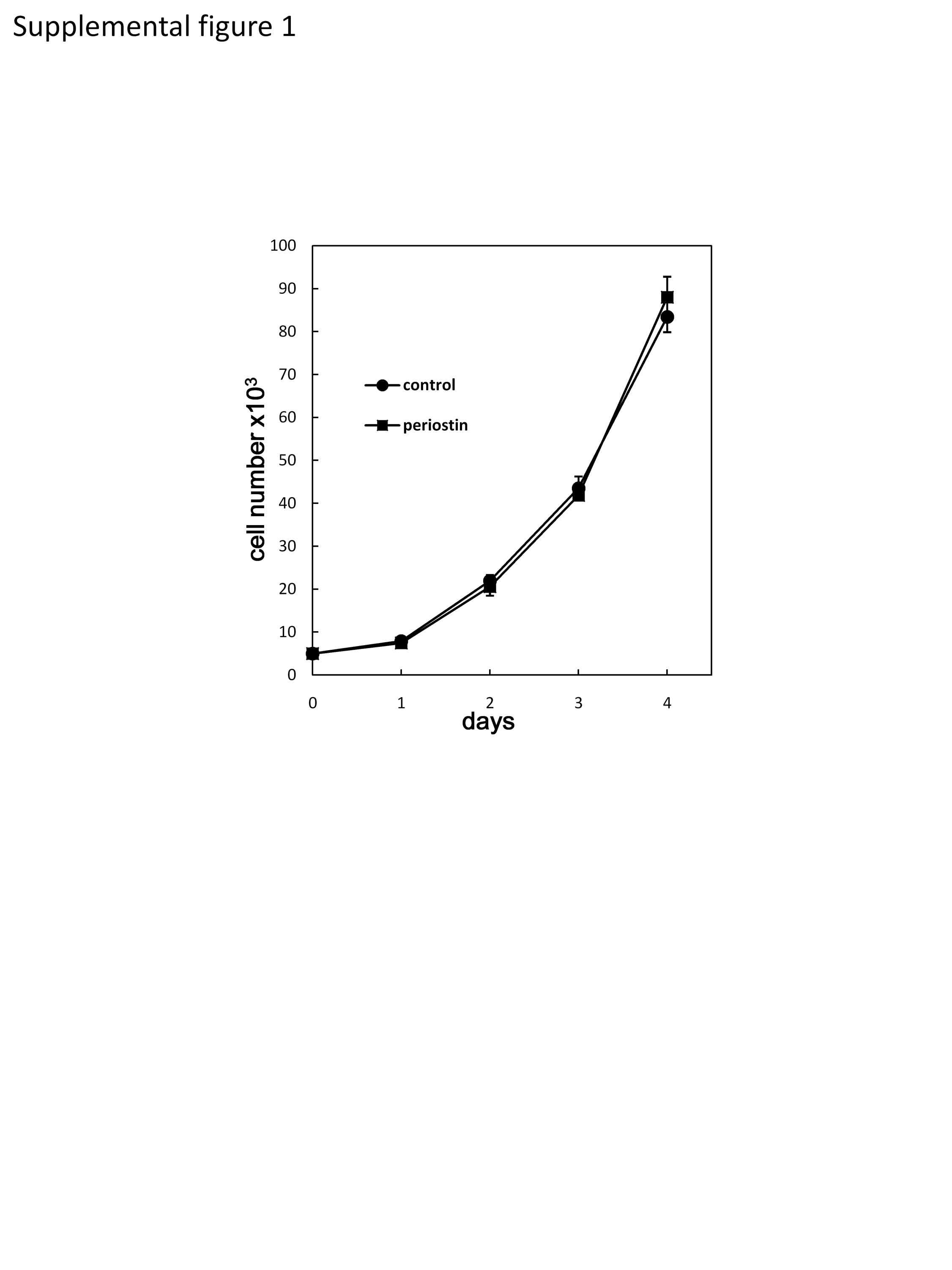

Supplement: Figure S1 — Proliferation of the periostin-HA HaCaT transfectant. The proliferation was compared between the transfectants produced with the periostin-HA or the vector only (control). (TIF) [file pone.0018410.s001.tif]

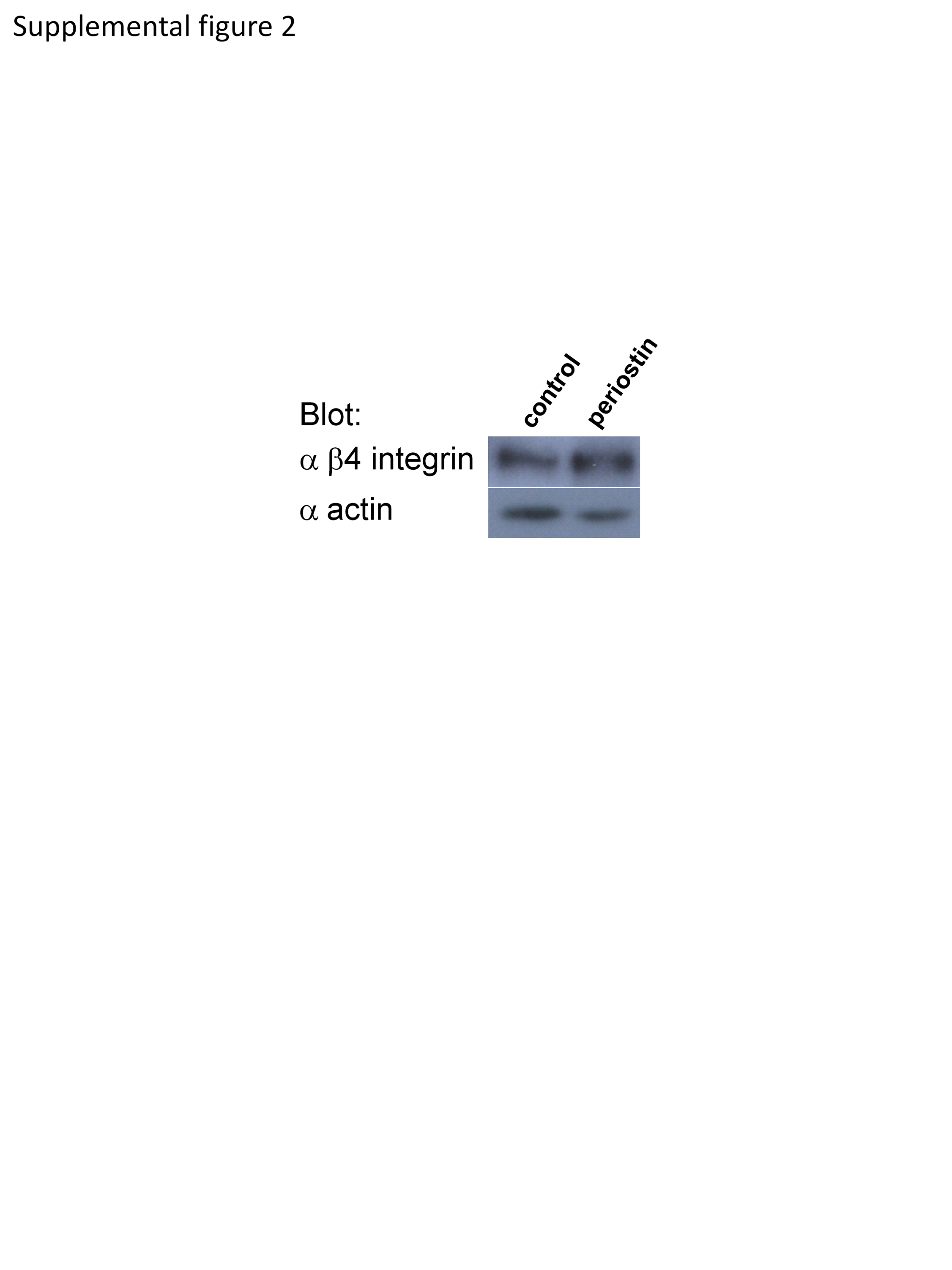

Supplement: Figure S2 — The β4 integrin expression in control and periostin-HA transfectants. Since NF-κB phosphorylation is dependent on the binding of β4 integrin and laminin 5, we examined the expression of β4 integrin, and observed the expression of β4 integrin in HaCaT cells; although no significant difference in expression was found in between control and periostin transfectants. (TIF) [file pone.0018410.s002.tif]
